# Dose effects of iron on growth, antioxidant potential, intestinal morphology, and intestinal barrier in yellow-feathered broilers

**DOI:** 10.1016/j.psj.2025.104865

**Published:** 2025-02-01

**Authors:** J. Chen, K.W. Lei, S.Y. Li, D.P. Li, Y.L. Wang, X. Wang, X. Bai, Y.L. Huang

**Affiliations:** a Key Laboratory of Animal Science of State Ethnic Affairs Commission, College of Animal and Veterinary Sciences, Southwest Minzu University*,* Chengdu 610041, PR China; b Key Laboratory of Qinghai-Tibetan Plateau Animal Genetic Resource Reservation and Utilization*,* College of Animal and Veterinary Sciences, Southwest Minzu University*,* Chengdu 610041, PR China

**Keywords:** Dose effect, Iron, Intestinal barrier, Yellow-feathered broiler

## Abstract

This experiment was conducted to investigate the dose effects of iron on growth performance, antioxidant function, small intestinal histology, and intestinal barrier of 63-day-old yellow-feathered broilers. A total of 720 1-day-old male yellow-feathered broilers were randomly divided into 9 treatments, with 8 cages per treatment and 10 birds per cage. The Fe supplementation was 0, 20, 40, 60, 80, 160, 320, 640, and 1280 mg/kg, respectively, in the form of FeSO_4_•7H_2_O. The results showed that the ADG (*P* = 0.002) and ADFI (*P* < 0.001) decreased linearly with increased dietary Fe supplementation. Malondialdehyde (**MDA**) concentration in plasma (*P* = 0.001), duodenum (*P* < 0.001), and jejunum (*P* < 0.001) were increased linearly as dietary Fe increased. As dietary Fe increased, there was a linear decrease in the villus height and the villus height/crypt depth in the duodenum (*P* = 0.003; *P* = 0.001) and jejunum (*P* = 0.001; *P* < 0.001). Decreased secretory immunoglobulin A (sIgA) concentration in jejunal mucosa (*P* < 0.001) was observed with increased dietary Fe concentration. Lower jejunal sIgA concentrations were observed in birds consuming more than 160 mg/kg of Fe (*P* < 0.001). A quadratic response was found for jejunal diamine oxidase (**DAO**) activity (*P* = 0.011) as dietary Fe supplementation was increased. The highest response of DAO in jejunal mucosa was observed for broilers supplemented with 160 mg/kg of Fe. Furthermore, the mRNA expressions of *ZO-1* (*P* < 0.001), *occludin* (*P* = 0.004), and *claudin-1* (*P* = 0.007) in jejunal mucosa decreased linearly with increased dietary Fe concentration. Data from the study suggests that there is no need to supplement additional Fe to a corn-soybean-based diet for yellow-feathered broilers based on growth performance, antioxidant potential, small intestinal histology, and intestinal barrier. Chronic iron exposure (≥ 160 mg/kg) can damage the intestinal barrier function, and further increase of Fe supplementation can lead to oxidative stress and even cause growth inhibition for yellow-feathered broilers.

## Introduction

The intestinal barrier is usually composed of mechanical, chemical, microbial, and immune barriers, and damage to the barrier will affect intestinal health, threatening the growth and health of animals. Evidence shows that mineral elements play an important role in intestinal barrier function (Wan et al., 2022). Iron deficiency promotes inflammatory responses in the intestinal mucosa and increases paracellular permeability, which in turn disrupts the intestinal barrier (Li et al., 2016). Excess Fe intake can also cause impairment of the intestinal barrier. In the model of mice, supplementation with 500 mg/kg Fe from FeSO_4_ causes intestinal histopathological damage and promotes colonization and migration of pathogens in weaning mice (Lin et al., 2018), and vitamin C exacerbates the damage of Fe to the intestinal barrier (Guo et al., 2020). Similar results were observed in the pig model. Iron overload disrupted the intestinal immune barrier in piglets of d 21 by promoting the expression of pro-inflammatory factors such as tumor necrosis factor α (**TNF-α**) and interleukin-1β (**IL-1β**) (Li et al., 2016) and changed the composition of the intestinal microbiome in piglets of d 21 (Chen et al., 2020). Similar results were found in broilers. Recently, we found that the supplementation of 320 mg/kg Fe in the diet impairs the intestinal barrier function of 21-day-old broilers (Lei et al., 2024). Therefore, the intake of appropriate amounts of Fe is vital for the intestinal health of animals.

In agricultural broiler production, dietary Fe supplementation is a standard practice to prevent iron deficiency. Broilers require 80 mg Fe/kg dry matter (DM) (NRC, 1994); however, many commercial diets contain amounts of Fe in considerable excess of this requirement. This is due to the fact that Fe is supplemented to the diet and the fact that several mineral feed ingredients, such as dicalcium phosphate and limestone, contain high concentrations of Fe (2,410-12,600 mg/kg) (Sullivan et al., 1994). The Fe in dicalcium phosphate and limestone was usually considered unavailable to animals. However, in a recent study, Fe in commercial limestone and dicalcium phosphate were found to meet the typical nutritional requirements of broilers (Feijo et al., 2024). Additional Fe supplementation, regardless of high Fe concentration in the feed ingredients, results in broilers consuming far more Fe than their requirements. The intake of excessive Fe poses not only a threat to animal health but also leads to an increase in Fe excretion, which subsequently leads to environmental pollution (La Carpia et al., 2019).

A considerable amount of research has focused on early age supplementation of Fe, based on the assumption that young animals have high Fe demand and are more susceptible to Fe deficiency (Forbes et al., 1972). However, animals are typically raised for extended periods in production, resulting in prolonged and continuous exposure to Fe. Studies with young pigs of different ages have provided some evidence that the expression of some genes involved in Fe metabolism was affected by age, suggesting age dependency on Fe metabolism (Hansen et al., 2010). Yellow-feathered broilers, whose production is comparable to that of white-feathered broilers in China, require a long rearing period to reach their market weight (from 63 days to 120 days). We recently examined the effect of Fe on the intestinal barrier in 21-day-old yellow-feathered broilers. However, the dose effects of prolonged iron exposure to yellow-feathered broilers are unclear. We propose the hypothesis that moderate or high doses of Fe may have positive or negative effects on growth performance and intestinal health, respectively, in 63-day-old yellow-feathered broilers.

Therefore, the present experiment was conducted to investigate the dose effects of iron on growth performance, antioxidant capacity, small intestinal histology, and intestinal barrier function of yellow-feathered broilers from 1 to 63 d of age, providing a theoretical basis and practical plan for the rational use of Fe in the production of yellow-feathered broilers.

## Materials and methods

### Animal, diets, and management

A total of 720 1-day-old male yellow-feathered broilers were selected and randomly divided into 9 treatments, with 8 cages per treatment and 10 birds per cage. The Fe supplementation in each treatment group was 0, 20, 40, 60, 80, 160, 320, 640, and 1280 mg/kg, respectively, in the form of FeSO_4_•7H_2_O. The treatment diets were prepared by mixing 0.64 % of a corn starch-FeSO_4_•7H_2_O premix with 99.36 % of a base mix. After blending the FeSO_4_•7H_2_O with corn starch, the resulting mixture provided supplemental Fe at concentrations of 0, 20, 40, 60, 80, 160, 320, 640, or 1280 mg/kg of diet. The corn-soybean meal basal diet was formulated to meet or exceed the nutritional requirements for broilers except for Fe (NRC, 1994; MAPRC, 2020). The formula and nutrient level of the basal diet are shown in Table 1. The analyzed Fe concentrations of different treatments are shown in Table 2. All the birds were housed in a temperature-controlled room with fiberglass feeders and stainless-steel cages coated with plastic. The initial temperature set point was 35.0 °C when chicks were placed in cages. The room temperature was maintained at 35 °C for one week and then reduced by 3 °C each week until the birds were 35 days old. Subsequently, the temperature was maintained at 22 °C until the end of the experiment. The birds had free access to feed and deionized water, and the light regimen was 24 h every day. The experiment lasted for 63 days and was divided into 3 stages: d 1∼21, d 22∼42, and d 43∼63.

**Table 1:** Composition of the basal diet for broilers (air-dry basis).

Ingredient	d 1-21	d 22-42	d 43-63
Corn (%)	56.83	64.78	67.70
Soybean meal (%)	35.75	28.45	23.98
Soybean oil (%)	3.00	2.50	4.30
NaCl (%)^1^	0.30	0.30	0.30
CaCO_3_ (%)^1^	1.00	0.92	0.91
CaHPO_4_ (%)^1^	1.84	1.70	1.53
Premix (%)^2^	0.22	0.22	0.22
L-Lys (%)	0.11	0.20	0.20
DL-Met (%)	0.23	0.26	0.20
L-Thr (%)	0.08	0.03	0.02
Corn starch-Fe premix (%)^4^	0.64	0.64	0.64
Total	100	100	100
Nutrition level			
Metabolic energy (Mcal/kg)^5^	2.97	3.01	3.15
Crude protein (%)^3^	21.33	18.66	16.78
Ca (%)^3^	0.94	0.96	0.80
Total P (%)^3^	0.70	0.73	0.60
Nonphytate P (%)	0.41	0.37	0.34
Lys (%)	1.18	1.08	0.96
Met (%)	0.53	0.53	0.45
Cys + Met (%)	0.84	0.80	0.72
Thr (%)	0.85	0.70	0.63
Fe (mg/kg)^3^	79.60	72.64	61.76
Cu (mg/kg)^3^	14.87	12.12	13.81
Mn (mg/kg)^3^	93.03	99.69	93.88
Zn (mg/kg)^3^	88.62	90.27	92.88

1 Reagent grade.

2 The premix provided the following diets per kilogram: Vitamin A 8,400 IU, vitamin D3 3,600 IU, vitamin E 13 IU, vitamin K 1.6 mg, thiamine 5.5 mg, vitamin B2 6.8 mg, vitamin B6 1.0 mg, vitamin B12 0.01 mg, biotin 0.08 mg, folic acid 0.80 mg, pantothenic acid 10.2 mg, niacin 28.6 mg, choline (choline chloride) 1,000 mg, Cu (as reagent grade blue copperas) 8 mg, Zn (as reagent grade zinc sulfate heptahydrate) 60 mg, Mn (as reagent grade manganese sulfate monohydrate) 80 mg, I (as feed grade calcium iodate) 0.35 mg, Se (as feed grade sodium selenite) 0.15 mg.

3 Analyzed values, and each value was based on triplicate determinations.

4 Fe supplements were added in place of equivalent weights of cornstarch in the form of FeSO_4_•7H_2_O.

5 When expressed in "MJ" units, the ME values are 12.45 MJ, 12.58 MJ, and 13.18 MJ, respectively.

**Table 2:** Dietary Fe concentrations (air-dry basis)^1^.

Age	Dietary Fe supplemental concentration (mg/kg)								
	0	20	40	60	80	160	320	640	1280
d 1-21	79.6	97.6	122	154	163	236	393	723	1354
d 22-42	72.6	91.3	116	134	151	229	388	700	1348
d 43-63	61.7	79.3	102	125	144	226	378	709	1412

1 The concentration of Fe in the diets was measured by flame atomic absorption spectrometry (Contr AA 700, Analytik Jena, Germany) after wet digestions with HNO_3_.

### Growth performance

Birds were weighted on d 1 and d 63; feed intake and number of deaths were recorded from d 1 to d 63, and dead individuals were weighted and recorded. The final body weight was obtained on d 63. The ADG per cage was calculated by subtracting the initial weight from the final weight and dividing it by the number of feeding days and eventually surviving birds. The ADFI per cage was corrected by subtracting the feed intake of dead birds from the total feed intake and dividing it by the number of feeding days and eventually surviving birds. The feed conversion ratio (**FCR**) was calculated by dividing the total feed intake by the total body weight gain per cage, including dead birds. Mortality was calculated by dividing the total number of deaths per cage by the total number of chickens per cage.

### Sample collection

On d 63, two birds close to the average weight were selected from each cage for sample collection. Blood was collected from the wing vein in a blood collection tube with sodium heparin and then centrifuged at 860 × g for 10 min at 4°C. The plasma was obtained and stored at −80°C for later analysis. After the collection of blood samples, birds were harvested by cervical dislocation. Segments (2-3 cm) of duodenum and jejunum were collected and fixed in a 4 % paraformaldehyde solution for histometric analysis. Then, duodenum and jejunum mucosa were immediately obtained after rinsing with PBS, frozen in liquid nitrogen, and stored at −80 °C for later analysis.

### Plasma and mucosal antioxidant potential

The activities of glutathione peroxidase (**GSH-Px**), total superoxide dismutase (**T-SOD**), and the concentration of malondialdehyde (**MDA**) in plasma, as well as T-SOD activity and MDA concentration in duodenal and jejunal mucosa, were detected by commercial kits (Nanjing Jiancheng Bioengineering Institute, Nanjing, China) following the instructions.

### Intestine morphological analysis

After 24 h of fixation, the intestinal tissue sections were made through paraffin embedding, sectioning, hematoxylin-eosin (**HE**) staining, and so on. The intestinal morphology was observed with a light microscope (DM1000, Leica, Germany) by selecting the intact villi and crypts. A total of 10 well-oriented villi were measured per section for their villus height (**VH**) and crypt depth (**CD**), and the ratio of villus height to crypt depth (**V/C**) was calculated.

### Jejunal secretory immunoglobulin a, diamine oxidase, and alkaline phosphatase assay

The concentration of secretory immunoglobulin A (**sIgA**) and the activity of alkaline phosphatase (**AKP**) and diamine oxidase (**DAO**) in the jejunal mucosa were detected by commercial kits (Nanjing Jiancheng Bioengineering Institute, Nanjing, China) following the instructions.

### Gene expression analysis

Based on the results of growth performance, antioxidant function, and intestinal morphology, the 0, 20, 80, 320, and 1280 mg/kg Fe supplementation groups were selected to analyze the mRNA expression of tight junction protein in jejunum mucosa. First, total RNA was extracted using a Trizol reagent (Takara, Dalian, China). Then, Nanodrop ND-1000 (Thermo Fisher, Waltham, USA) was used to analyze the purity of RNA (OD 260/280 ≥ 1.8), and the integrity of RNA was determined by gel electrophoresis. Reverse transcription was performed using the PrimeScript™ RT Reagent Kit (TaKaRa, Ltd., Dalian, China). The resulting cDNA was diluted 8-fold and used as a PCR template for real-time qPCR analysis in the CFX Connect^TM^ RealTime PCR Detection System (Hercules, CA). The reaction system had a volume of 25 μL and contained 12.5 μL TB Green Premix Ex Taq II (Takara, Dalian, China), 2 μL cDNA, 1 μL upstream primer, 1 μL downstream primer, and 8.5 μL ddH_2_O. Primers were synthesized by Shanghai Sangu Biotechnology Co., LTD., and the sequence of primers is shown in Table 3. In addition, the PCR parameters were as follows: 30 s at 95 °C, 40 cycles at 95 °C for 5 s, and 60 °C for 30 s. All reactions were performed in triplicate, and the expressions of relative mRNA were determined by 2^−ΔΔCT^method normalized with the level of *β-actin*.

**Table 3:** Primer sequences of real-time fluorescence quantitative PCR.

Gene	GenBank Accession No.	Primer Sequence (5′- 3′)	Amplicon Size (bp)
*β-actin*	NM_205518.2	F: CGGTACCAATTACTGGTGTTAGATG	163
		R: GCCTTCATTCACATCTATCACTGG	
*ZO-1*	XM_046925214.1	F: CCGCAGTCGTTCACGATCT	63
		R: GGAGAATGTCTGGAATGGTCTGA	
*Occludin*	XM_046904540.1	F: GATGGACAGCATCAACGACC	193
		R: CATGCGCTTGATGTGGAAGA	
*Claudin-1*	NM_001013611.2	F: TCGGGCCTTCTATGACCCTT	176
		R: GGGCATTTTTGGGGTAGCCT	

### Statistical analysis

Data analysis was performed by one-way ANOVA using the MIXED procedure of SAS (SAS Inst. Inc., Cary, NC) with cage as the experimental unit. The LSMEANS procedure of SAS was used to calculate treatment means, and the PDlFF option was used to separate means if the difference was significant. Data from the mortality of broilers were transformed using Arcsin before statistical analysis. Orthogonal comparisons (unequally spaced) were applied for linear and quadratic responses. Significance was determined at *P* < 0.05.

## Results

### Growth performance

Growth performance data are presented in Table 4. A nonsignificant effect on the average initial body weight (*P* = 1.000), FCR (*P* = 0.330), and mortality (*P* = 0.266) was observed. However, the average final body weight (*P* < 0.001), ADG (*P* = 0.002), and ADFI (*P* < 0.001) of yellow-feathered broilers showed a linear response to increased dietary Fe supplementation. Compared to the control group, the diet supplemented with 1280 mg/kg Fe significantly decreased the average final body weight (*P* < 0.001), ADG (*P* < 0.001), and ADFI (*P* < 0.001) of yellow-feathered broilers.

**Table 4:** Effects of dietary supplemental Fe on growth performance of yellow-feathered broilers^1^.

Item	Dietary Fe supplemental concentration (mg/kg)									SEM	*P-*value		
	0	20	40	60	80	160	320	640	1280		Treatment	Linear	Quadratic
IBW (g)	46.65	46.65	46.65	46.66	46.66	46.65	46.65	46.65	46.65	0.129	1.000	0.991	0.996
FBW (g)	2446.81^a^	2422.97^a^	2382.23^a^	2413.69^a^	2393.23^a^	2402.84^a^	2414.20^a^	2367.15^a^	2153.61^b^	14.638	<0.001	<0.001	0.030
ADG (g)	38.98^a^	38.46^a^	37.93^a^	38.38^a^	38.04^a^	38.31^a^	38.47^a^	38.37^a^	34.11^b^	0.240	<0.001	<0.001	0.002
ADFI (g)	82.68^ab^	80.33^a^	82.61^ab^	81.70^ab^	82.48^ab^	85.90^b^	81.46^abc^	81.23^ab^	71.11^c^	0.643	<0.001	<0.001	<0.001
FCR (g/g)	2.11	2.12	2.18	2.13	2.17	2.21	2.12	2.15	2.09	0.012	0.330	0.174	0.299
Mortality (%)	5.71	3.75	3.75	5.00	5.00	4.29	2.50	0.00	3.75	0.631	0.266	0.334	0.073

1 IBW, average initial body weight; FBW, average final body weight; ADG, average daily body weight gain; ADFI, average daily feed intake; FCR, feed conversion ratio. (*n* = 8). ^a,b^ means in a row not sharing a common superscript letter differ (*P* < 0.05) and the same below.

### Antioxidant potential

Plasma and intestinal antioxidant properties of yellow-feathered broilers are shown in Table 5. The concentration of MDA in plasma (*P* = 0.001), duodenal (*P* < 0.001), and jejunal mucosa (*P* < 0.001) was linearly increased with the increase of Fe supplementation. Compared to the control, 320 mg/kg of Fe supplementation or greater increased the concentration of MDA in the duodenum (*P <* 0.001) and jejunum mucosa (*P <* 0.001). In addition, linear response was detected for T-SOD activity in the jejunum (*P* < 0.001). Birds supplemented with more than 80 mg/kg of Fe had a lower jejunal T-SOD activity than those in the control group (*P* = 0.003). However, dietary Fe supplementation had a nonsignificant effect on the activities of GSH-Px in plasma (*P* = 0.935), as well as T-SOD in plasma (*P* = 0.133) and duodenal mucosa (*P* = 0.483).

**Table 5:** Effects of dietary supplemental Fe on antioxidant capacity of yellow-feathered broilers^1^.

Item	Dietary Fe supplemental concentration (mg/kg)									SEM	*P-*value		
	0	20	40	60	80	160	320	640	1280		Treatment	Linear	Quadratic
Plasma													
GSH-Px (U/mL)	3025.66	2865.81	3582.20	3368.99	3514.64	4053.52	3692.69	3365.20	3795.31	198.833	0.935	0.502	0.724
T-SOD (U/mL)	874.55	872.93	943.88	987.26	994.69	806.84	992.07	979.15	950.83	18.33	0.133	0.337	0.315
MDA (nmol/mL)	1.88^a^	2.53^a^	2.15^a^	2.32^a^	1.96^a^	2.55^a^	3.08^ab^	3.07^ab^	4.05^b^	0.157	0.029	0.001	0.090
Duodenum													
T-SOD (U/mgprot)	332.13	331.98	312.58	296.26	313.83	319.27	297.88	287.36	278.76	6.409	0.483	0.032	0.443
MDA (nmol/mgprot)	0.09^b^	0.09^b^	0.08^b^	0.12^b^	0.12^b^	0.13^b^	0.19^a^	0.24^a^	0.22^a^	0.008	<0.001	<0.001	0.011
Jejunum													
T-SOD (U/mgprot)	384.32^a^	400.25^a^	345.55^ab^	334.78^ab^	278.57^b^	299.00^b^	299.67^b^	263.73^b^	296.47^b^	9.850	0.003	<0.001	0.129
MDA (nmol/mgprot)	0.10^c^	0.11^c^	0.14^bc^	0.15^bc^	0.17^bc^	0.18^bc^	0.21^b^	0.31^a^	0.31^a^	0.013	<0.001	<0.001	0.087

1 Each mean represents an average of 8 cages with 2 broilers sampled per cage. (*n* = 8). GSH-Px, glutathione peroxidase; T-SOD, total superoxide dismutase; MDA, malondialdehyde. T-SOD and MDA were protein-corrected in the mucosa of the duodenum and jejunum.

### Intestinal morphology

**Fig. 1:**
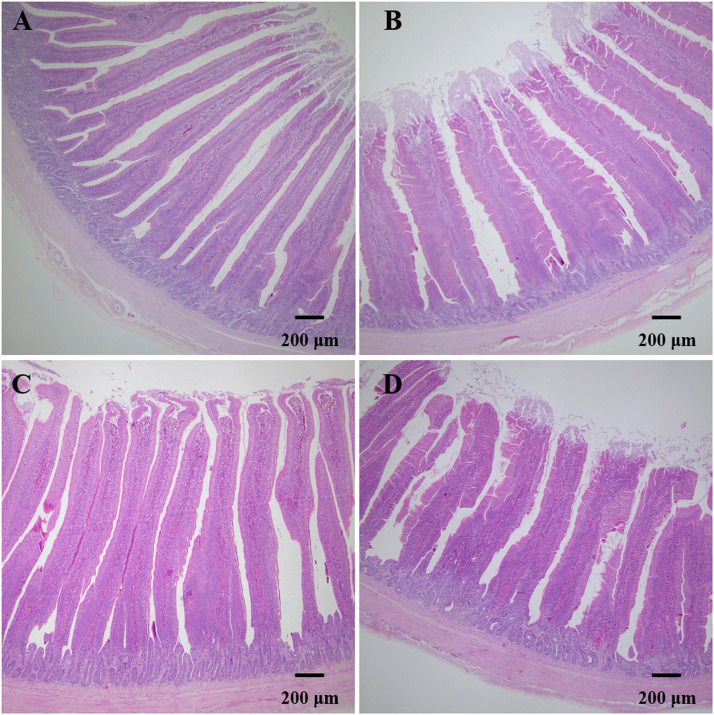
Fe changes the intestinal morphology of yellow-feathered broilers. A, duodenum morphological structure in the control diet; B, duodenum morphological structure in the diet supplemented with 1280 mg/kg of Fe; C, jejunum morphological structure in control diet; D, jejunum morphological structure in the diet supplemented with 1280 mg/kg of Fe.

The morphology and structure of the duodenum and jejunum of yellow-feathered broilers are shown in Fig. 1 and Table 6. As shown in Fig. 1 and Table 6, the VH in the duodenum (*P* < 0.001) and jejunum (*P* < 0.001) had a linear relationship with dietary Fe supplementation. Compared to the control group, dietary Fe supplementation of 640 mg/kg or higher decreased the VH of the duodenum (*P* = 0.003), whereas, in the jejunum, the significance was observed when supplemented with more than 160 mg/kg of Fe (*P* = 0.001). Besides, the V/C was decreased linearly both in the duodenum (*P =* 0.002) and jejunum (*P <* 0.001), and lower V/C was detected for birds supplemented with 640 mg/kg or greater of Fe both in the duodenum (*P =* 0.001) and jejunum (*P <* 0.001), respectively. However, a nonsignificant effect on the CD of the duodenum (*P* = 0.123) and jejunum (*P* = 0.056) was observed.

**Table 6:** Effect of dietary supplemental Fe on intestinal villus height and crypt depth of yellow-feathered broilers^1^.

Item	Dietary Fe supplemental concentration (mg/kg)									SEM	*P*-value		
	0	20	40	60	80	160	320	640	1280		Treatment	Linear	Quadratic
Duodenum													
VH (mm)	1855.85^a^	1847.27^a^	1773.48^ab^	1829.97^ab^	1775.90^ab^	1722.07^abc^	1720.09^abc^	1687.59^bc^	1609.79^c^	16.751	0.003	<0.001	0.384
CD (mm)	157.81	181.99	181.09	167.01	172.68	164.01	145.51	189.49	199.82	4.406	0.123	0.043	0.231
V/C (mm/mm)	12.19^a^	10.87^a^	10.18^ab^	12.02^a^	11.83^a^	11.23^a^	12.77^a^	7.50^b^	7.77^b^	0.368	0.001	0.002	0.013
Jejunum													
VH (mm)	1563.79^a^	1487.29^ab^	1538.88^ab^	1461.12^abc^	1420.72^abc^	1382.93^bcd^	1394.11^bcd^	1321.49^cd^	1258.05^d^	19.167	0.001	<0.001	0.546
CD (mm)	162.48	162.14	172.38	159.09	181.75	153.16	154.28	204.80	200.97	4.801	0.056	<0.001	0.360
V/C (mm/mm)	9.16^a^	9.56^a^	8.61^ab^	9.40^a^	8.21^ab^	9.50^a^	8.38^ab^	7.06^bc^	6.01^c^	0.228	<0.001	<0.001	0.012

1 Each mean represents an average of 8 cages with 2 broilers sampled per cage. (*n* = 8). VH, villus height; CD, crypt depth; V/C, the ratio of villus height to crypt depth.

### Concentrations of intestinal sIgA and activity of intestinal diamine oxidase and alkaline phosphatase

As shown in Table 7, with the increase of dietary Fe supplementation, the concentration of sIgA in the jejunal mucosa showed a linear decrease (*P* < 0.001). Compared to the control, the concentration of sIgA decreased when dietary Fe supplementation was 160 mg/kg or greater (*P* = 0.001). Furthermore, a quadratic response was detected for the activity of jejunal DAO as dietary Fe supplementation was increased (*P* = 0.011). The highest response of DAO activity in the jejunal mucosa was observed for broilers fed a diet supplemented with 160 mg/kg of Fe. However, a nonsignificant effect on the activity of AKP in the jejunum was observed (*P* = 0.507).

**Table 7:** Effects of dietary supplemental Fe on the activity of sIgA/AKP/DAO in the jejunal mucosa of yellow-feathered broilers^1^.

Item	Dietary Fe supplemental concentration (mg/kg)									SEM	*P*-value		
	0	20	40	60	80	160	320	640	1280		Treatment	Linear	Quadratic
sIgA (µg/mgprot)	8.38^a^	8.17^a^	8.29^a^	6.04^ab^	5.92^abc^	4.78^bc^	4.15^bc^	4.88^bc^	2.71^c^	0.399	0.001	<0.001	0.994
AKP (U/gprot)	29.96	35.93	28.95	29.61	30.71	26.04	25.61	26.30	27.31	1.138	0.507	0.227	0.145
DAO (U/mgprot)	12.29^bc^	16.83^ab^	18.91^a^	16.58^ab^	16.36^ab^	19.28^a^	16.16^ab^	11.09^c^	14.53^abc^	0.593	0.011	0.457	0.002

1 Each mean represents an average of 8 cages with 2 broilers sampled per cage. (*n* = 8). sIgA, secreted immunoglobulin A; AKP, alkaline phosphatase; DAO, diamine oxidase.

### Relative mRNA expression of tight junction proteins

**Fig. 2:**
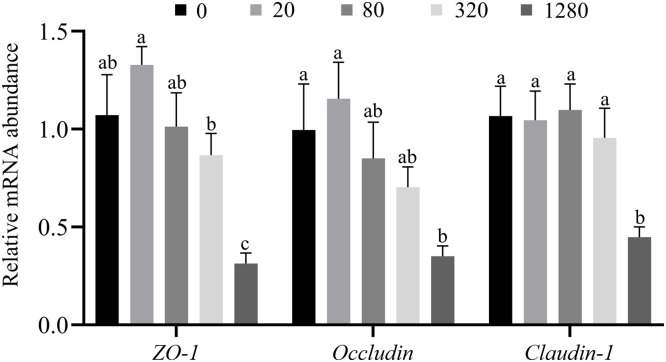
Effect of dietary supplemental Fe concentration on mRNA expression of tight junction protein in jejunal mucosa of yellow-feathered broilers. 0, control; 20, a diet supplemented with 20 mg/kg of Fe; 80, a diet supplemented with 80 mg/kg of Fe; 320, a diet supplemented with 320 mg/kg of Fe; 1280, a diet supplemented with 1280 mg/kg of Fe. Each mean represents an average of 8 cages with 2 broilers sampled per cage. (*n* = 8).

The mRNA expression of tight junction protein in the jejunal mucosa of yellow-feathered broilers is shown in Fig. 2. As shown in Fig. 2, the mRNA expressions of *ZO-1* (*P* < 0.001), *occludin* (*P* = 0.004), and *claudin-1* (*P* = 0.007) decreased linearly with the increased dietary Fe supplementation. Compared to other groups, the addition of 1280 mg/kg of Fe reduced the gene expressions of *ZO-1* (*P* < 0.001), *occludin* (*P* = 0.031)*,* and *claudin-1* (*P* = 0.016) in the jejunal mucosa of yellow-feathered broilers.

## Discussion

The results of the current study partly supported our hypothesis that dietary high Fe concentration had a negative effect on growth performance, oxidant capacity, small intestine histology, and intestinal barrier of yellow-feathered broilers, while a positive effect on low Fe supplementation was not observed. Young animals have a higher Fe requirement compared to adult animals, making them more susceptible to iron deficiency (Forbes et al., 1972). As a result, most research on Fe has focused on young animals or the early stages of growth (Fang et al., 2018; Liu et al., 2023; Li et al., 2016; Guo et al., 2018). Studies in our group have found that adding low doses of 20 mg Fe/kg to the basal diet improved the growth performance of 21-day-old yellow-feathered broilers (Lei et al., 2024). However, no improvement in growth performance with low doses of Fe addition was observed in 1-63d yellow-feathered broilers, suggesting that animals at different stages of growth have different reactions to Fe supplementation.

In the present study, ADFI and ADG of yellow-feathered broiler of 1-63 d were linearly decreased with increased dietary Fe supplementation. Decreased feed intake may have a relationship with the pro- and anti-feeding hormones secreted by the gastrointestinal tract and adipose tissue (Ranft et al., 1991; Iovino et al., 2022). It has been found that a certain amount of iron exposure could reduce fat accumulation (Kim et al., 2022; Lei et al., 2022) and cause damage to the intestinal tract (Guo et al., 2018; Lei et al., 2024), which may in turn affect the pro- and anti-feeding hormones secretion, and cause the decrease of ADFI. Due to the decreased feed intake and damaged intestinal tract, the ADG of the broilers decreased.

An imbalance in homeostasis between oxidative and antioxidant oxidation in the body leads to oxidative stress, resulting in organismal damage (Sies, 1997). Iron is involved in single electron transfer reactions and is essential for forming and metabolizing oxygen radicals in vivo. It has been shown that Fe absorbed in animals is mainly bound to transferrin. When the Fe binding capacity of transferrin is fully saturated, the remaining non-transferrin-bound iron (**NTBI**) is deposited in the liver, heart, and endocrine system, where it catalyzes Fenton and Haber-Weiss oxidation of lipids, resulting in the formation of hydroxyl and other derived radicals (Jomova et al., 2022). Malondialdehyde is the final product of lipid peroxidation and reflects the body's antioxidant capacity and the extent of cellular damage. In the current study, MDA concentrations in plasma, duodenum, and jejunum of 63 d yellow-feathered broilers were increased linearly as dietary Fe increased. Similar results were obtained by other researchers. Gou et al. (2018) found dietary supplementation with 700 mg/kg (measured 908 mg/kg) of Fe elevated the concentration of MDA in the jejunal mucosa of 21-day-old yellow-feathered broilers. Studies in our group also observed increased mucosal MDA in 21-day-old yellow-feathered broilers when 640 mg/kg of Fe was supplemented (Lei et al., 2024). However, different results were obtained in trials with varying days of age. It was noted that supplementation with 800 mg/kg of Fe did not change the concentration of MDA in the liver of 42-day-old AA broilers (Han et al., 2022). In the study at 21 days of age, the concentration of MDA in mucosal increased when supplemented with 640 mg/kg of Fe (Lei et al., 2024), whereas in the 63-day-old study, the result was 320 mg/kg. Studies have found that the activity of SOD increases with age, and the redox homeostasis of young animals is more susceptible to external influences (Nesterova et al., 2024). Therefore, the young animals are believed to be more vulnerable to oxidative stress. In addition, it has been found that excess Fe decreases mitochondrial aldehyde dehydrogenase activity, resulting in the accumulation of MDA in the organism (Britton et al., 1990). These findings suggest that iron overload may promote lipid peroxidation and interfere with MDA metabolism, leading to the accumulation of MDA in tissues and causing greater damage to young animals.

The GSH-Px and T-SOD are necessary antioxidant enzymes in vivo, which have the function of scavenging oxygen free radicals and other physiological functions in the body, and their activity reflects the antioxidant ability of the organism. Studies have shown that excess Fe inhibits the biosynthesis of glutathione and glutathione peroxidase 4 (Gou et al., 2018; Cao et al., 2016) and reduces the activity of T-SOD (Han et al., 2022). The results of the present study revealed that the activity of T-SOD in jejunal mucosa decreased linearly with increasing dietary Fe supplementation. Total-SOD comprises SOD1 (Cu/Zn-SOD), SOD2 (Mn-SOD), and SOD3 (EC-SOD). Research has demonstrated that Fe absorption exhibits an antagonistic effect with that of copper (Cu) and manganese (Mn) because of competition for common intestinal transporters (Einhorn et al., 2024). Copper and Mn serve as essential cofactors for SOD1 and SOD2, respectively (Zelko et al., 2002). As a result, a reduction of copper and manganese can impair the T-SOD activity (Fakhri et al., 2024). This may partly explain the reason for decreased T-SOD activity with increased dietary Fe supplementation. Few changes in T-SOD activity were observed in the mucosa and plasma of 21-day-old yellow-feathered broilers (Lei et al., 2024). This may be due to different Fe metabolism of animals at different ages (Friese et al., 2024). Hansen et al. (2010) reported that the expression of some genes involved in Fe metabolism was affected by age. Therefore, prolonged and continuous iron exposure may affect T-SOD activity through interactions that influence trace element absorption. Overall, iron overload predisposes animals to oxidative stress in the intestine and compromises their intestinal health.

Intestinal mucosal development is an ongoing process driven by the continuous proliferation, migration, and differentiation of intestinal stem cells (**ISCs**) in the crypts. The dietary composition plays a crucial role in influencing this developmental process (Duangnumsawang et al., 2021). Research has shown that excess Fe inhibits the proliferation and differentiation of ISCs by suppressing the Notch signaling pathway (Zhao et al., 2024), with both factors synergistically affecting the renewal and integrity of intestinal epithelial cells (**IECs**). Moreover, the NTBI catalyzes the Fe-dependent Fenton reaction, generating a large amount of lipid peroxidation products, which may induce ferroptosis in cells (Galaris et al., 2019). Studies have found that excessive Fe intake negatively impacts the development of intestinal mucosal, with age-dependent differences in dose response (Luo et al., 2020; Li et al., 2016). Gou et al. (2018) reported that adding 700 or 1,400 mg/kg Fe to a broiler diet decreased the VH of the jejunum at 21 days of age. Lei et al. (2024) obtained similar results at 21 days of age (1,280 mg/kg in the duodenum and 640 mg/kg in the jejunum). However, lower dietary Fe supplemented doses (640 mg/kg in the duodenum and 160 mg/kg in the jejunum), which changed the intestinal morphology of 63-day-old yellow-feathered broilers, were observed in the current study. These findings indicate that prolonged and continuous iron exposure may occur at a lower dose of dietary Fe supplementation.

Secretory immunoglobulin A (**sIgA**), as an important part of the intestinal immune barrier, has been consistently recognized as an essential regulator in maintaining the intestinal barrier (Johnson et al., 1999; Nakamura et al., 2020). Studies have shown that the concentration of intestinal sIgA is age-dependent, with its expression decreasing as age progresses (Branca et al., 2019). A prolonged iron exposure (16 weeks) has been shown to reduce the concentration of sIgA in the intestinal mucosa of 35-week-old mice (Luo et al., 2020). In the present study, the dietary Fe supplementation of 63-day-old yellow-feathered broilers linearly decreased the concentration of intestinal sIgA, which may be related to the disruption of the intestinal mucosal structure and inflammation mediated by iron overload. In addition, AKP, located at the intestinal mucosa's brush border, maintains tight junction proteins between intestinal mucosal cells. It inhibits the expression of pro-inflammatory factors and TNF-α induced by lipopolysaccharides, the absence of which leads to an increase in the intestinal inflammatory response (Singh et al., 2021). It has been shown that piglets injected with excess Fe (900 mg) exhibited a significant reduction in serum AKP activity (Li et al., 2011). However, we found that the activity of AKP in the intestinal mucosa was not affected by the concentration of Fe in the diet. The observed discrepancies may be attributed to factors such as species differences, variations in Fe administration methods, and the physiological stage of the animal (Kühn et al., 2021). These findings suggest that high Fe intake may impair the intestinal immune barrier by lowering the concentration of sIgA in the intestinal mucosa.

The intestinal mechanical barrier is an intestinal epithelial barrier composed mainly of columnar epithelial cells and the connections between cells. The intestinal epithelium maintains its selective barrier function by forming a complex network of proteins that connects epithelial cells containing four types of adhesion complexes: desmosomes, adherens junctions, gap junctions, and tight junction proteins (Groschwitz et al., 2009; Huo et al., 2022). The tight junction proteins provide substantial barriers to macromolecules (Johnson et al., 1999). Defects in the tight junction proteins lead to increased intestinal permeability, promoting the development of intestinal inflammation and Inflammatory Bowel Disease (Huo et al., 2022). It has been found that oxidative stress induced by iron exposure disrupts the tight junction proteins of the intestinal epithelium and increases intestinal permeability (Huo et al., 2022). Studies have shown that prolonged iron exposure (16 weeks) reduces the mRNA and protein expression of tight junction proteins ZO-1, occludin, and claudin-1 in the jejunum of 35-week-old mice in a dose-dependent manner (Luo et al., 2020). However, the phenomenon of asynchronous gene and protein expression may arise, and the underlying cause of this discrepancy could be the variation in the duration of iron exposure. Fang et al. (2018) found that short-term (30 days) oral administration of 24 mg of Fe to 4-week-old rats did not change the protein expression of ZO-1, occludin, and claudin-1. Similarly, a study on weaned piglets at 23 days of age showed that short-term (28 days) Fe exposure (300 mg/kg) did not change the protein expression of tight junction proteins ZO-1, occludin, and claudin-1 in the duodenum (Ding et al., 2020). Additionally, short-term (3 h) high iron exposure (100 μM) did not induce changes in the morphology of tight junction protein ZO-1 in Caco-2 cells (Ferruzza et al., 2002). Our findings reveal a dose-dependent difference in the mRNA expression of tight junction proteins between 21-day and 63-day-old animals. This discrepancy suggests that further analysis of protein expression may be necessary to elucidate this issue. In conclusion, these findings suggest that iron overload may regulate the mRNA and protein expression of tight junction proteins by influencing the demethylation of genes encoding these proteins (Wang et al., 2023), and this change likely requires an accumulation of iron exposure duration.

In addition, DAO, as an intracellular enzyme secreted by intestinal cells, is a sensitive indicator for evaluating the function of the intestinal mechanical barrier (Wolvekamp et al., 1994). It can break down diamines such as histamine. When the body is subjected to stress injury, the intestinal permeability is altered, and DAO is released from the mucosal cells into the bloodstream, resulting in a decrease in the activity of DAO in the intestine and an increase in the activity of DAO in the bloodstream (Liu et al., 2020). Plasma DAO activity is considered a sensitive indicator for assessing damage to the intestinal physical barrier (Huo et al., 2022). In studies of intestinal barrier function, DAO in plasma is believed to originate from IECs. Research has shown that prolonged and continuous iron exposure (12 weeks, 1000 mg/kg) increases the concentration of DAO in the plasma of rats (Ma et al., 2018). Our present research showed that the activity of DAO in the jejunum mucosa changed quadratically as the Fe supplementation was increased. When supplemented with more than 160 mg/kg of Fe, the activity of DAO in the jejunum showed a tendency to decrease, indicating that the intestinal mechanical barrier was damaged above that level.

Recent studies have found that commercial limestone and dicalcium phosphate contain sufficient Fe for broiler growth (Feijo et al., 2024). The mineral additives used in this experiment were reagent grade to avoid interference by impurity Fe, and the drinking water was deionized so that the Fe ingested by the animals was derived from the Fe contained in the natural raw materials and FeSO_4_•7H_2_O used as an additive. The results showed that the basal diets composed of corn, soybean meal, and reagent-grade additives contained sufficient Fe for growth performance and intestinal health of yellow-feathered broilers, indicating relatively high bioavailability of Fe in natural ingredients such as corn and soybean meal. Moreover, more than 160 mg/kg of additional Fe supplementation showed a negative effect on the growth performance and intestinal barrier function of yellow-feathered broilers in the present study. These results indicated no additional Fe is needed for yellow-feathered broilers fed with a commercial broiler diet due to the relatively high Fe bioavailability of commercial mineral additives (limestone and dicalcium phosphate) and natural ingredients. However, the bioavailability of Fe in natural ingredients may be affected by some external factors, such as the origin and year of production. The bioavailability of Fe in ingredients of broiler diets needs to be further investigated and clarified.

## Conclusion

The basal diet without Fe supplementation has sufficient Fe for normal growth and intestinal health of yellow-feathered broilers. Chronic iron exposure (≥ 160 mg/kg of Fe) reduced the growth performance of 63-day-old yellow-feathered broilers, induced oxidative stress in vivo, and destroyed the mechanical and immune barrier functions of the intestinal tract.

## Disclosures

We declare that we have no financial and personal relationships with other people or organizations that can inappropriately influence our work, and there is no professional or other personal interest of any nature or kind in any product, service, company, or both that could be construed as influencing the content of this paper.

## Declaration of competing interest

We declare that we have no financial and personal relationships with other people or organizations that can inappropriately influence our work, and there is no professional or other personal interest of any nature or kind in any product, service, company, or both that could be construed as influencing the content of this paper.
